# Multimodal Regional Anesthesia Combining Spinal and Erector Spinae Plane Block for Spine Surgery in a High-Risk Patient with Systemic Sclerosis: A Case Report

**DOI:** 10.5812/aapm-160051

**Published:** 2025-03-29

**Authors:** Alireza Shakeri, Jina Behjati

**Affiliations:** 1 Anesthesiology Research Center, Shahid Beheshti University of Medical Sciences, Tehran, Iran; 2 School of Medicine, Shahid Beheshti University of Medical Sciences, Tehran, Iran

**Keywords:** Case Report, Erector Spinae Plane Block (ESPB), Spinal Anesthesia (SA), Spine Surgery, Systemic Sclerosis

## Abstract

**Introduction:**

The erector spinae plane block (ESPB) is a novel regional anesthesia technique that is increasingly incorporated into multimodal analgesia as part of enhanced recovery after surgery (ERAS) pathways in various surgical procedures, including spine surgery.

**Case Presentation:**

We report the successful use of spinal anesthesia (SA), ESPB, and magnesium sulfate in a high-risk patient with systemic sclerosis and pulmonary fibrosis undergoing laminectomy. A multimodal approach was selected due to the patient’s underlying condition. This strategy minimized respiratory complications associated with general anesthesia while providing effective surgical anesthesia and postoperative pain control without opioid-related complications.

**Conclusions:**

Our case highlights the utility of ESPB, not only for postoperative pain management but also as a valuable adjunct to primary anesthesia, especially in high-risk patients.

## 1. Introduction

The erector spinae plane block (ESPB), first described by Forero et al., is an innovative regional anesthetic technique that delivers local anesthetics into the interfascial plane between the erector spinae muscles and transverse processes (1). This approach provides a multi-dermatomal sensory block of the anterior, posterior, and lateral thoracic and abdominal walls (1). The ESPB has been widely utilized across various surgeries, including spine surgery (2-5), due to its technical simplicity, safety profile, and effective pain control (6-8). Multiple systematic reviews have demonstrated the benefits of ESPB in spine surgery, such as reducing opioid consumption, decreasing postoperative nausea and vomiting, and shortening hospital stays (8-10). While ESPB is commonly used as an adjunct to general anesthesia (GA) (11-13) or as a postoperative analgesic technique (14), its potential as part of a multimodal regional anesthesia in the context of open spine surgery remains largely unexplored, with only one case documented in the literature (15). This is particularly important when trying to avoid GA-associated complications in high-risk patients (16).

In this report, we present our experience using a multimodal regional anesthetic approach — spinal anesthesia (SA) and ESPB with magnesium sulfate infusion — in a patient with systemic sclerosis and pulmonary fibrosis undergoing laminectomy. This case highlights the potential advantages of regional anesthesia techniques in managing a high-risk patient while minimizing perioperative risks and optimizing patient outcomes.

## 2. Case Presentation

**Figure 1.**
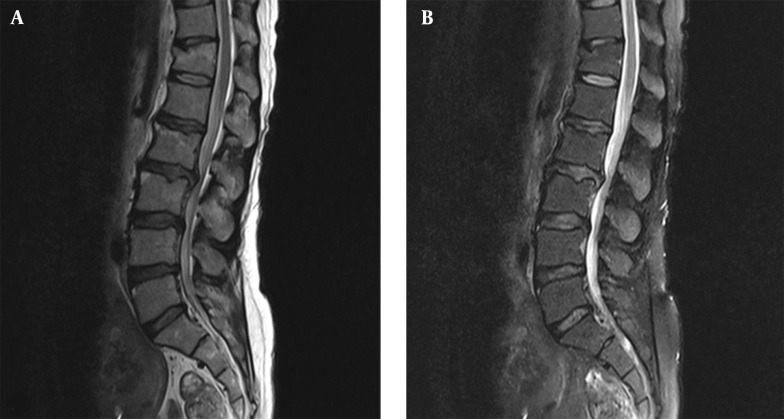
A, T1 weighted; and B, T2 weighted magnetic resonance imaging of the lumbar spine indicating canal stenosis at levels L2-L4

**Figure 2.**
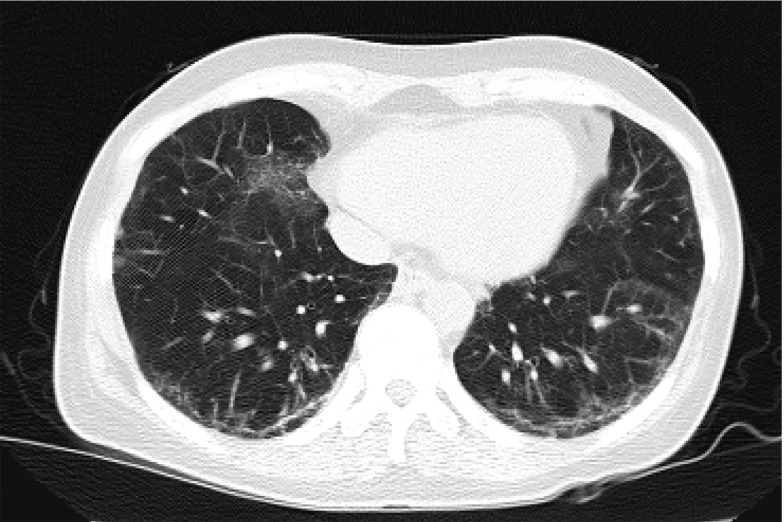
Computed tomography (CT) scan of the lungs showing diffuse reticular opacities and traction bronchiectasis characteristic of pulmonary fibrosis

A 46-year-old woman (ASA III) presented with progressive back pain radiating to her left foot, which did not respond to medical management and necessitated surgical intervention. Her medical history was significant for systemic sclerosis with pulmonary fibrosis and pulmonary arterial hypertension, as well as hypothyroidism. The patient had limited mouth opening due to dermal thickening. On neurological examination, she had normal muscle strength and intact reflexes. MRI of the spine revealed spinal stenosis at levels L2-L4 (Figure 1). A chest CT scan showed pulmonary fibrosis (Figure 2), and pulmonary function tests demonstrated a restrictive pattern. Additionally, her systolic pulmonary artery pressure was measured at 40 mmHg, necessitating minimal sedation due to cardiopulmonary concerns.

Given the patient's medical history, which placed her at high risk for respiratory complications associated with general anesthesia, we decided to adopt a regional anesthetic approach. Standard monitoring was implemented, including heart rate, non-invasive blood pressure, pulse oximetry, side stream capnography, electrocardiography, and temperature to ensure hemodynamic stability throughout the surgery.

**Figure 3.**
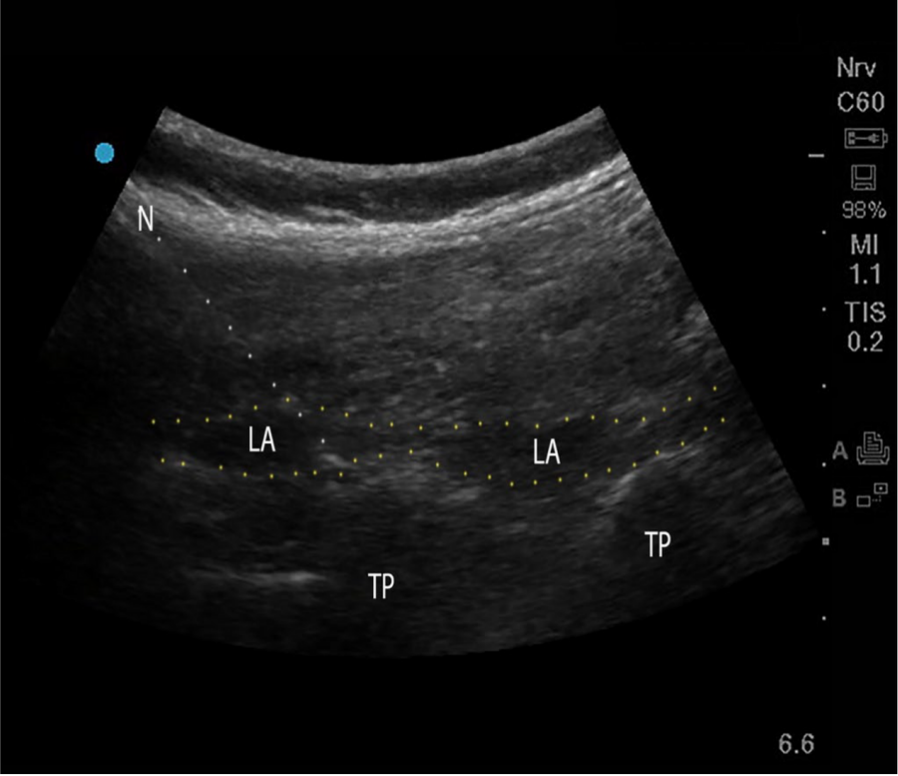
Ultrasound guided lumbar erector spinae plane block (ESPB); Abbreviations: TP, transverse process; LA, local anesthetic; N, needle.

The ESPB was performed under ultrasound guidance using a curvilinear transducer (3 - 8 MHz, Sonosite Edge II; Sonosite, Inc., FUJIFILM). The transducer was positioned to visualize the L3 transverse process. Under sterile conditions, a 22-gauge, 80 mm needle (B. Braun Stimuplex Ultra 360) was advanced craniocaudally using the in-plane approach until the needle tip was in the fascial plane between the erector spinae muscle and the transverse process of the L3 vertebra. A total of 40 mL of 0.25% ropivacaine was injected bilaterally (Figure 3). 

Following ESPB, spinal anesthesia was administered at the L3 level. Using a 25-gauge spinal needle, 3 mL of 0.5% bupivacaine was injected into the subarachnoid space after confirming clear cerebrospinal fluid flow. As an adjunct to the regional anesthesia, intravenous magnesium sulfate was loaded and maintained at a rate of 30 mg/kg/h throughout the surgery.

The patient underwent L2-L4 laminectomy to alleviate the spinal stenosis. The surgery took 2.5 hours and was completed without perioperative complications. In the post-anesthesia care unit (PACU), the patient reported no pain (visual analog scale score of 0). The patient was transferred to the neurosurgical ward, where analgesia was first required 12 hours after surgery. Following the ward's standard analgesic protocol, the patient received acetaminophen, gabapentin, and oxycodone at fixed intervals, with diclofenac available as rescue medication. Diclofenac was only needed on postoperative days 2 and 3. The patient was discharged on postoperative day 4.

## 3. Discussion

We present the anesthetic management of a high-risk patient with systemic sclerosis who underwent lumbar laminectomy using regional anesthesia — SA and ESPB — plus magnesium sulfate infusion. The ESPB in open spine surgery is typically added to general anesthesia or used as postoperative analgesia, making our case the second report of its kind in the literature to use ESPB with spinal anesthesia. This multimodal approach provided both intraoperative and postoperative advantages. Intraoperatively, adequate anesthesia was achieved while avoiding general anesthesia (GA)-associated complications and maintaining hemodynamic stability. Postoperatively, it resulted in sufficient pain control, reduced opioid consumption, and no cardiopulmonary complications or postoperative nausea and vomiting. Spine surgery patients typically experience severe pain in the first 12 hours postoperatively, which intensifies with movement due to surgical trauma and tissue damage (13). With this approach, our patient remained pain-free during this period post-surgery. This is especially important for systemic sclerosis patients as they are more sensitive to respiratory depression associated with traditional opioid-based pain management (13, 17).

Systemic sclerosis is an autoimmune disease characterized by progressive fibrosis of the skin and internal organs such as the heart, lungs, kidneys, and gastrointestinal system (18). The anesthesia approach for these patients must be customized based on the specific organs affected by this condition. Our patient had interstitial pulmonary fibrosis and pulmonary artery hypertension. Additionally, she had dermal thickening and limited mouth opening, which made tracheal intubation challenging. Despite general anesthesia being the standard approach for open spine surgery (19, 20), given our patient’s condition, we opted against it to minimize the risks of potential respiratory complications (16) and adopted a regional anesthesia (RA) approach.

In spine surgery, RA is primarily achieved through either epidural or spinal anesthesia (21). Both techniques, while effective, require careful consideration. Key challenges include the lack of a secure airway, technical difficulties, and potential interference with neuromonitoring (21). In our case, we utilized spinal anesthesia as our primary anesthetic technique. Spinal anesthesia has demonstrated comparable safety and efficacy to GA, with the added benefit of prolonged analgesic effects leading to faster recovery and discharge (22). Although spinal anesthesia alone is considered sufficient for laminectomy (23), we incorporated ESPB and magnesium sulfate into our anesthetic regimen. This decision was primarily influenced by our center's extended operative time of 2.5 hours, compared to typical durations of 79 and 97 minutes for SA and GA, respectively (24). This extended duration was due to our setting as a teaching hospital involving resident training. Managing adequate anesthesia for this prolonged period using SA alone would have required higher doses of local anesthetics, potentially causing hemodynamic instability — a particular concern in our patient.

In a similar approach, Karthik et al. demonstrated the efficacy of combined thoracic segmental SA and ESPB as a safe and effective alternative to general anesthesia in high-risk patients with multiple comorbidities (hepatic, renal, and cardiac), providing hemodynamic stability, superior analgesia, quicker recovery, and early ambulation (25). The ESPB is well-established in spine surgery and has consistently demonstrated improved pain control, lower opioid requirement, enhanced recovery, and patient satisfaction (8, 14). It also enhances surgical conditions via muscle relaxation and provides longer postoperative analgesia, lasting up to 72 hours, ensuring a smoother transition from the intraoperative to postoperative period (22). However, in the context of open spine surgery, it is either used in combination with general anesthesia or in postoperative pain management (8, 14, 26). To our knowledge, there is only one previous record describing the combination of ESPB with spinal anesthesia used for a dual minimally invasive transforaminal lumbar interbody fusion and lumbar decompression in an 87-year-old patient, who tolerated the procedure without any postoperative complications (15). This makes our case particularly valuable in advancing regional anesthesia approaches for open spine surgery. It is noteworthy that while ESPB has been used as the sole anesthetic approach in some cases (27-29), it has not been reported for open spine surgery.

The ESPB typically involves large volumes of local anesthetic, with optimal amounts in adults ranging from 20 - 30 mL (30). Our use of 20 mL of 0.25% ropivacaine bilaterally (40 mL total) aligns with these recommendations. This technique allows the anesthetic to spread within the fascial plane over 3 - 6 vertebral levels in a craniocaudal direction, providing effective multi-level analgesia while ensuring doses remain within safe limits to avoid systemic toxicity (30). In a recent meta-analysis, ESPB was compared to thoracolumbar interfascial plane (TLIP) block and midtransverse process to pleura (MTP) block in lumbar spine surgery, and there were no significant differences in postoperative opioid consumption, pain scores at 24 hours, or incidence of postoperative nausea and vomiting between ESPB and TLIP block (9). However, ESPB has a simpler execution and fewer complications due to its target and needle trajectory being distant from critical structures such as pleura and major vessels (7).

We added intravenous magnesium sulfate infusion into our anesthetic approach due to its well-documented advantage in reducing post-surgery pain, required doses of anesthetic medication, opioid consumption, and postoperative nausea and vomiting (31-34). Magnesium achieves its analgesic effect by blocking NMDA receptors, thereby reducing pain transmission and central nervous system excitability (35). Also, it acts as a calcium channel blocker, leading to bronchodilation and vasodilation in arterioles and coronary arteries (34). Its antiarrhythmic properties help prevent potential bupivacaine-associated tachyarrhythmias, enhancing overall patient safety and outcomes (34). In our study, intraoperative hemodynamics remained stable, with mean arterial pressure decreases limited to less than 20% of initial values without requiring vasopressors, consistent with a similar study in patients undergoing posterior lumbar spinal fusion surgery that measured heart rate and mean arterial pressure without significant differences between magnesium and control groups (36).

In conclusion, our case demonstrated the successful implementation of multimodal regional anesthesia, combining SA, ESPB, and magnesium sulfate infusion for lumbar laminectomy in a high-risk patient with systemic sclerosis. Our experience suggests that this multimodal strategy could be an alternative to general anesthesia for similar high-risk patients undergoing spine surgery, especially when prolonged operative times are anticipated. Our findings represent a single case, and previous studies on this combination have been limited; therefore, future larger prospective studies are needed to establish its role in routine clinical practice.

## Data Availability

Data sharing is not applicable to this article as it is a case report and does not involve any datasets.

## References

[1] Forero M, Adhikary SD, Lopez H, Tsui C, Chin KJ (2016). The Erector Spinae Plane Block: A Novel Analgesic Technique in Thoracic Neuropathic Pain.. Reg Anesth Pain Med..

[2] Ueshima H, Inagaki M, Toyone T, Otake H (2019). Efficacy of the Erector Spinae Plane Block for Lumbar Spinal Surgery: A Retrospective Study.. Asian Spine J..

[3] Tulgar S, Kapakli MS, Senturk O, Selvi O, Serifsoy TE, Ozer Z (2018). Evaluation of ultrasound-guided erector spinae plane block for postoperative analgesia in laparoscopic cholecystectomy: A prospective, randomized, controlled clinical trial.. J Clin Anesth..

[4] Bhushan S, Huang X, Su X, Luo L, Xiao Z (2022). Ultrasound-guided erector spinae plane block for postoperative analgesia in patients after liver surgery: A systematic review and meta-analysis on randomized comparative studies.. Int J Surg..

[5] Leong RW, Tan ESJ, Wong SN, Tan KH, Liu CW (2021). Efficacy of erector spinae plane block for analgesia in breast surgery: a systematic review and meta-analysis.. Anaesthesia..

[6] Kot P, Rodriguez P, Granell M, Cano B, Rovira L, Morales J (2019). The erector spinae plane block: a narrative review.. Korean J Anesthesiol..

[7] Sethuraman RM (2024). Erector spinae plane block in spine surgeries: Single-level versus bi-level, single-shot versus continuous catheter technique.. Saudi J Anaesth..

[8] Oh SK, Lim BG, Won YJ, Lee DK, Kim SS (2022). Analgesic efficacy of erector spinae plane block in lumbar spine surgery: A systematic review and meta-analysis.. J Clin Anesth..

[9] Liu H, Zhu J, Wen J, Fu Q (2023). Ultrasound-guided erector spinae plane block for postoperative short-term outcomes in lumbar spine surgery: A meta-analysis and systematic review.. Med (Baltimore)..

[10] Liu MJ, Zhou XY, Yao YB, Shen X, Wang R, Shen QH (2021). Postoperative Analgesic Efficacy of Erector Spinae Plane Block in Patients Undergoing Lumbar Spinal Surgery: A Systematic Review and Meta-Analysis.. Pain Ther..

[11] Avis G, Gricourt Y, Vialatte PB, Meunier V, Perin M, Simon N (2022). Analgesic efficacy of erector spinae plane blocks for lumbar spine surgery: a randomized double-blind controlled clinical trial.. Reg Anesth Pain Med..

[12] Subbiah M, Madhuvarshinee KM, Vinothan RJS, Poornima V, Ramanarayanan R, Manikandan B (2023). A Novel Combined Anesthetic Technique to Improve the Surgical Working Conditions of Lumbar and Thoracolumbar Spine Surgery from a Spine Surgeon's Perspective: A Prospective Randomized Controlled Study.. Asian Spine J..

[13] Zhu J, Wu Z, Huang G, Zhong Y, Peng C (2023). Effect of Erector Spinae Plane Block in Terms of Analgesic Efficacy in Elderly Patients Undergoing Posterior Lumbar Spine Surgery: A Retrospective, Propensity-Score Matched Study.. Pain Ther..

[14] Rizkalla JM, Holderread B, Awad M, Botros A, Syed IY (2021). The erector spinae plane block for analgesia after lumbar spine surgery: A systematic review.. J Orthop..

[15] Wilson JP, Bonin B, Quinones C, Kumbhare D, Guthikonda B, Hoang S (2024). Spinal Anesthesia for Awake Spine Surgery: A Paradigm Shift for Enhanced Recovery after Surgery.. J Clin Med..

[16] Efrimescu CI, Donnelly S, Buggy DJ (2023). Systemic sclerosis. Part II: perioperative considerations.. BJA Educ..

[17] Dinges HC, Otto S, Stay DK, Baumlein S, Waldmann S, Kranke P (2019). Side Effect Rates of Opioids in Equianalgesic Doses via Intravenous Patient-Controlled Analgesia: A Systematic Review and Network Meta-analysis.. Anesth Analg..

[18] Carr ZJ, Klick J, McDowell BJ, Charchaflieh JG, Karamchandani K (2020). An Update on Systemic Sclerosis and its Perioperative Management.. Curr Anesthesiol Rep..

[19] Zorrilla-Vaca A, Healy RJ, Mirski MA (2017). A Comparison of Regional Versus General Anesthesia for Lumbar Spine Surgery: A Meta-Analysis of Randomized Studies.. J Neurosurg Anesthesiol..

[20] De Rojas JO, Syre P, Welch WC (2014). Regional anesthesia versus general anesthesia for surgery on the lumbar spine: a review of the modern literature.. Clin Neurol Neurosurg..

[21] Lee JK, Park JH, Hyun SJ, Hodel D, Hausmann ON (2021). Regional Anesthesia for Lumbar Spine Surgery: Can It Be a Standard in the Future?. Neurospine..

[22] Kang TH, Kim WJ, Lee JH (2023). Efficacy of the erector spinae plane block with sedation for unilateral biportal endoscopic spine surgery and comparison with other anesthetic methods.. Acta Neurochir (Wien)..

[23] Baenziger B, Nadi N, Doerig R, Proemmel P, Gahl B, Hodel D (2020). Regional Versus General Anesthesia: Effect of Anesthetic Techniques on Clinical Outcome in Lumbar Spine Surgery: A Prospective Randomized Controlled Trial.. J Neurosurg Anesthesiol..

[24] Urick D, Sciavolino B, Wang TY, Gupta DK, Sharan A, Abd-El-Barr MM (2022). Perioperative outcomes of general versus spinal anesthesia in the lumbar spine surgery population: A systematic review and meta-analysis of data from 2005 through 2021.. J Clin Orthop Trauma..

[25] Karthik GS, Chandra M, Sudheer R, Shwetha AH (2025). Combined thoracic segmental spinal anesthesia and erector spinae plane block in high-risk patients undergoing thoracoscopic surgery: A case series.. Saudi J Anaesth..

[26] Liang X, Zhou W, Fan Y (2021). Erector spinae plane block for spinal surgery: a systematic review and meta-analysis.. Korean J Pain..

[27] Shakeri A, Memary E (2024). Erector spinae plane block as an anesthesia technique for an emergent thoracotomy; a case report.. BMC Anesthesiol..

[28] Cardoso TM, Viegas C, Amaral E, Sá M, Torgal R, Caramelo S (2025). Erector Spinae Plane Block as an Anesthetic Technique for Open Gastrostomy: A Case Report.. Cureus..

[29] Tulgar S, Yildirim A, Karaoglan A, Ozer Z (2019). Erector spinae plane block as the main anesthetic method for peri-paravertebral area surgical procedure.. J Clin Anesth..

[30] Chin KJ, Lirk P, Hollmann MW, Schwarz SKW (2021). Mechanisms of action of fascial plane blocks: a narrative review.. Reg Anesth Pain Med..

[31] Hwang JY, Na HS, Jeon YT, Ro YJ, Kim CS, Do SH (2010). I.V. infusion of magnesium sulphate during spinal anaesthesia improves postoperative analgesia.. Br J Anaesth..

[32] Forget P, Cata J (2017). Stable anesthesia with alternative to opioids: Are ketamine and magnesium helpful in stabilizing hemodynamics during surgery? A systematic review and meta-analyses of randomized controlled trials.. Best Pract Res Clin Anaesthesiol..

[33] Do SH (2013). Magnesium: a versatile drug for anesthesiologists.. Korean J Anesthesiol..

[34] Roscoe A, Ahmed AB (2003). A survey of peri‐operative use of magnesium sulphate in adult cardiac surgery in the UK.. Anaesthesia..

[35] Woolf CJ, Thompson SWN (1991). The induction and maintenance of central sensitization is dependent on N-methyl-D-aspartic acid receptor activation; implications for the treatment of post-injury pain hypersensitivity states.. Pain..

[36] Dehkordy ME, Tavanaei R, Younesi E, Khorasanizade S, Farsani HA, Oraee-Yazdani S (2020). Effects of perioperative magnesium sulfate infusion on intraoperative blood loss and postoperative analgesia in patients undergoing posterior lumbar spinal fusion surgery: A randomized controlled trial.. Clin Neurol Neurosurg..

